# Effect of Angiotensin II Type 2 Receptor-Interacting Protein on Adipose Tissue Function via Modulation of Macrophage Polarization

**DOI:** 10.1371/journal.pone.0060067

**Published:** 2013-04-02

**Authors:** Fei Jing, Masaki Mogi, Li-Juan Min, Kousei Ohshima, Hirotomo Nakaoka, Kana Tsukuda, Xiaoli Wang, Jun Iwanami, Masatsugu Horiuchi

**Affiliations:** Department of Molecular Cardiovascular Biology and Pharmacology, Ehime University Graduate School of Medicine, Ehime, Japan; Osaka University Graduate School of Medicine, Japan

## Abstract

We demonstrated that angiotensin II type 2 (AT_2_) receptor-interacting protein (ATIP) 1 ameliorates inflammation-mediated vascular remodeling independent of the AT_2_ receptor, leading us to explore the possibility of whether ATIP1 could exert anti-inflammatory effects and play a role in other pathophysiological conditions. We examined the possible anti-inflammatory effects of ATIP1 in adipose tissue associated with amelioration of insulin resistance. In mice fed a high-cholesterol diet, adipose tissue macrophage (ATM) infiltration and M1-to-M2 ratio were decreased in ATIP1 transgenic mice (ATIP1-Tg) compared with wild-type mice (WT), with decreased expression of inflammatory cytokines such as tumor necrosis factor-α and monocyte chemoattractant protein-1 in white adipose tissue (WAT), but an increase in interleukin-10, an anti-inflammatory cytokine. Moreover, 2-[^3^H]deoxy-d-glucose (2-[^3^H]DG) uptake was significantly increased in ATIP1-Tg compared with WT. Next, we examined the roles of ATIP1 in BM-derived hematopoietic cells, employing chimeric mice produced by BM transplantation into irradiated type 2 diabetic mice with obesity, KKAy, as recipients. ATM infiltration and M1-to-M2 ratio were decreased in ATIP1 chimera (ATIP1-tg as BM donor), with improvement of insulin-mediated 2-[^3^H]DG uptake and amelioration of inflammation in WAT. Moreover, serum adiponectin concentration in ATIP1 chimera was significantly higher than that in WT chimera (WT as BM donor) and KKAy chimera (KKAy as BM donor). These results indicate that ATIP1 could exert anti-inflammatory effects in adipose tissue via macrophage polarization associated with improvement of insulin resistance, and ATIP1 in hematopoietic cells may contribute to these beneficial effects on adipose tissue functions in type 2 diabetes.

## Introduction

Proteins interacting with G protein coupled receptors have been highlighted as factors regulating these receptors. Accordingly, we have cloned ATIP angiotensin type 2 (AT_2_) receptor interacting protein (ATIP) [1], also known as AT_2_ receptor binding protein (ATBP) [2], as a protein interacting with the C-terminal tail of this receptor, using a yeast two-hybrid system. ATIP1 was found to be identical to a ubiquitously expressed tumor suppressor protein localized in mitochondria [3]. ATIP is also named mitochondrial tumor suppressor gene 1 (MTUS1), which shows mutation or copy number variants in human malignant tumors [3], [4], [5]. We investigated the role of ATIP1 in vascular remodeling using ATIP1 transgenic mice (ATIP1-Tg) [6]. We demonstrated that neointimal formation following inflammation-associated vascular injury of the femoral artery, which was induced by polyethylene cuff placement around the artery, was significantly smaller, with decreased superoxide anion production and expression of proinflammatory cytokines compared to those in WT littermates. Interestingly, pretreatment with PD123319, an AT_2_ receptor antagonist, could not prevent the reduction of neointimal formation, and superoxide anion production induced by not only angiotensin II but also thrombin was attenuated in vascular smooth muscle cells prepared from ATIP1-Tg [6]. These results suggest that ATIP1 plays an important role in pathological conditions via an AT_2_ receptor-independent mechanism. These results led us to examine the possibility that ATIP1 could act as an AT_2_ receptor-independent mechanism in other pathological conditions associated with inflammation.

A chronic low-grade inflammatory state in obesity plays a role in systemic metabolic dysfunction associated with obesity-linked disorders [7], [8]. Inflammation in white adipose tissue (WAT) is known to contribute to the pathogenesis of insulin resistance and type 2 diabetes [9], [10]. Although the detailed mechanisms are not fully understood, there is accumulating interest in the role of adipose tissue macrophages (ATM) as a player in inflammation in obesity [11], [12]. ATM consist of at least two different phenotypes: classically activated M1 macrophages and alternatively activated M2 macrophages. Lumeng et al. reported that ATM isolated from lean animals expressed hallmarks of polarization toward M2 macrophages with IL-10 and arginase expression, whereas in obese animals with insulin resistance, monocyte chemoattractant protein-1 (MCP-1) released from obese WAT caused a shift to M1-polarized ATM with increased TNF-αα and inducible nitric oxide synthase (iNOS) [13]. Diet-induced obesity leads to a shift in the activation state of ATM from an M2-polarized state in lean animals to an M1-polarized state, resulting in insulin resistance [13]. Fujisaka et al. also demonstrated that increases in M1 polarization and M1-to-M2 ratio are closely associated with insulin resistance induced by a high-fat diet [14]. However, factors that trigger the phenotypic switch of macrophage polarization are not well known.

Variation in the cellular cholesterol level induces changes in the inflammatory status of macrophages, and accumulation of cholesterol in macrophages is central to foam cell formation and the development of atherosclerosis [15]. Recently, Subramanian et al. reported that the addition of 0.15% cholesterol in the diet resulted in a marked increase in accumulation of macrophages in WAT [16]. Furthermore, an overload of cholesterol enhanced macrophage infiltration into WAT, thereby giving rise to inflammation and resulting in impaired insulin sensitivity [17]. From these results, we investigated the possibility of whether ATIP1 could ameliorate inflammation and thereby improve insulin sensitivity in WAT, focusing on macrophage infiltration and polarization using ATIP1-Tg fed a high-cholesterol diet. We also examined the roles of ATIP1 in hematopoietic cells, focusing on inflammation in WAT, employing irradiated type 2 diabetic mice with obesity, KKAy, as recipients of bulk bone marrow cell (BMC) transplantation.

## Materials and Methods

### Animals and Treatment

This study was performed in accordance with the National Institutes of Health guidelines for the use of experimental animals. All animal studies were reviewed and approved by the Animal Studies Committee of Ehime University.

Adult male mice carrying the mouse ATIP1-myc (ATIP1-Tg) gene as described previously were used [6]. Moreover, adult male wild-type littermate mice (WT) and obese type 2 diabetic model mice, KKAy (CLEA, Tokyo, Japan), were used. Animals were kept in a room in which lighting was controlled (12 hours on, 12 hours off) and temperature was kept at 25°C. In experiment 1, ATIP1-Tg and WT were fed a high-cholesterol diet [HCD: 1.25% cholesterol and 10% coconut oil in MF (Oriental Yeast Co. Ltd., Tokyo, Japan)] from 10 weeks of age. All experiments using ATIP1-Tg and WT were performed at 28 weeks of age.

### Quantitative RT-PCR

Expression of mRNA prepared from epididymal and retroperitoneal WAT was quantified by real-time RT-PCR with a SYBR Premix Ex Taq (Takara Bio Inc., Shiga, Japan) and primers as follows: 5′-CGAGTGACAAGCCTGTAGCC-3′ (forward) and 5′-GGTGAGGAGCACGATGTCG-3′ (reverse) for TNF-α, 5′-TTAACGCCCCACTCACCTGCTG-3′ (forward) and 5′-GCTTCTTTGGGACACCTGCTGC-3′ (reverse) for MCP-1, 5′-GCTCTTACTGACTGGCATGAG-3′ (forward) and 5′-CGCAGCTCTAGGAGCATGTG-3′ (reverse) for IL-10, and 5′-ATGTAGGCCATGAGGTCCAC-3′ (forward) and 5′-TGCGACTTCAACAGCAACTC-3′ (reverse) for glyceraldehyde-3-phosphate dehydrogenase (GAPDH).

### Glucose Uptake in Adipose Tissue

Glucose uptake in WAT was measured as the rate constant of 2-[^3^H]deoxy-d-glucose (2-[^3^H]DG) uptake according to our previous report [18]. Epididymal and retroperitoneal WAT were rapidly dissected and weighed. The rate constant of adipose tissue uptake of 2-[^3^H] DG was calculated as described previously [19].

### Isolation of Stromal-vascular Fraction from Adipose Tissue

Epididymal WAT from mice was digested with collagenase (2 mg/ml) (Sigma-Aldrich, St. Louis, MO) with Krebs-Henseleit–HEPES buffer (Nacalai Tesque, Tokyo, Japan) supplemented with 20 mg/ml bovine serum albumin (Sigma-Aldrich) and 2 mmol/l glucose (Sigma-Aldrich) at 37°C with shaking for 20 min. Then, the samples were passed through a cell strainer (BD, Franklin Lakes, NJ) and fractionated by centrifugation at 1000 rpm as previously described [14]. Cells in the pallets were collected as the stromal-vascular fraction (SVF) for flow cytometric analysis.

### Flow Cytometric Analysis

SVF were incubated with antibodies as follows: anti-mouse F4/80-FITC (eBioscience, San Diego, CA), CD11c-PE (eBioscience) and CD206-Alexa Fluor 647 (AbD Serotec, Oxford, UK) or the matching control isotypes (eBioscience) for 30 min at 4°C following incubation with 2.4G2 (BD) for 10 min. After incubation with 7-amino-actinomycin D (BD), the cells were analyzed with a Gallios (Beckman Coulter, Brea, CA). M1 macrophages were identified as F4/80-positive/CD11c-positive/CD206-negative and M2 macrophages as F4/80-positive/CD11c-negative/CD206-positive, according to a previous report [14].

### Generation of Chimeric Mice

In experiment 2, to analyze the functional role of ATIP in hematopoietic cells, we generated chimeric mice as described previously with minor modification [20]. Briefly, 8-week-old male KKAy were exposed to a total dose of 8 Gy whole-body irradiation and used as recipients. Bone marrow cells (BMC) were isolated from six crushed bones (bilateral tibias, femurs, and iliac bones) from 8-week-old male KKAy, WT or ATIP1-Tg. Bulk BMC (1.0×10^6^ cells) diluted in PBS (200 µL) were injected via the tail vein immediately after irradiation. Six weeks after transplantation, mice were used for the following experiments. To determine the chimerism, expression of ATIP1 in BMC was assessed by immunoblotting as shown in **Figure S1B**.

### Serum Adiponectin and TNF-α Concentrations

Blood samples were obtained at 9 to 10 a.m. by the submandibular bleeding method using an animal bleeding lancet (Goldenrod, Medipoint, Inc., NY) from mice after fasting for 16 hours. Serum adiponectin and TNF-α were measured using a Mouse/Rat High Molecular Weight Adiponectin ELISA kit (AKMAN-011, Shibayagi Co. Ltd., Gunma, Japan) and Mouse TNF-α ELISA kit (AKMTN-011, Shibayagi Co. Ltd.) according to the manufacturer’s protocol.

### Immunohistochemical Staining

To assess localized macrophage clusters surrounding adipocytes, paraffin-embedded sections of epididymal WAT fixed with 10% formalin were immunostained with an antibody against F4/80. Briefly, endogenous peroxidase was blocked by incubation in 3% H_2_O_2_ for 15 min, and nonspecific protein binding was blocked by incubation for 10 min in Blocking Reagent (Nichirei Bioscience Inc., Tokyo, Japan). Then, the sections were incubated with monoclonal anti-F4/80 antibody (diluted 1∶100) (BMA Biomedicals, August, Switzerland) followed by Histofine Simple Stain Max PO (Nichirei Bioscience Inc., Tokyo, Japan). Antibody binding was visualized with 3,3′-diaminobenzidine (DAB) using a detection kit (Nichirei Bioscience Inc., Tokyo, Japan), and all sections were counterstained with hematoxylin. Samples were examined with a Zeiss Axioskop2 microscope (Carl Zeiss, Oberkochen, Germany) equipped with a computer-based imaging system.

### Statistical Analysis

All values are expressed as mean ± SEM in the text and figures. Data were evaluated by ANOVA followed by post hoc analysis for multiple comparisons. A difference with P<0.05 was considered significant.

## Results

### Decrease in WAT Weight and Adipocyte Hypertrophy in ATIP-1Tg

**Figure 1:**
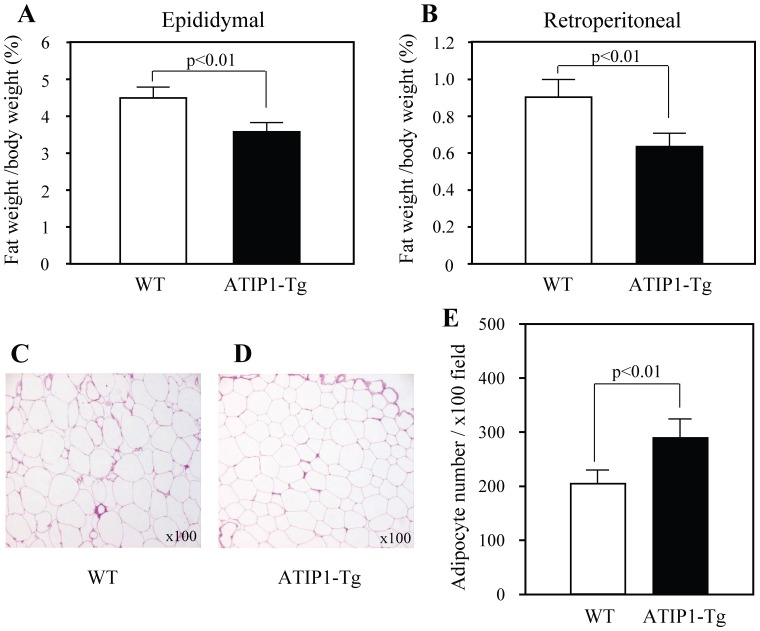
WAT in ATIP1-Tg and WT after treatment with high-cholesterol diet for 18 weeks. (**A**) Ratio of WAT weight to body weight in epididymal and retroperitoneal tissue. (**B**) Morphological comparison of epididymal WAT. Representative photomicrographs at ×100 magnification and histogram of adipocyte number per field. n = 7–8 for each group.

HCD was introduced at 10 weeks of age in ATIP1-Tg and WT. The ratio of abdominal WAT weight to body weight was more markedly increased by treatment with HCD from ND in WT (18.53±1.28% increase) than in ATIP-Tg (14.65±1.36% increase). Dietary intake did not differ between mice (2.8±0.2 g/day in ATIP1-Tg, 2.9±0.3 g/day in WT). The ratio of WAT weight to body weight both in epididymal and retroperitoneal tissue was significantly lower in ATIP1-Tg at 28 weeks of age compared with WT (**Figure 1A**). Histological examination of WAT showed that adipocyte size was smaller in ATIP1-Tg than in WT (**Figure 1B**). Accordingly, adipocyte number per field at ×100 magnification was markedly increased in ATIP1-Tg compared with WT (**Figure 1B**).

### Decrease in Proinflammatory Cytokine Levels and Increase in Glucose Uptake in ATIP1-Tg

**Figure 2:**
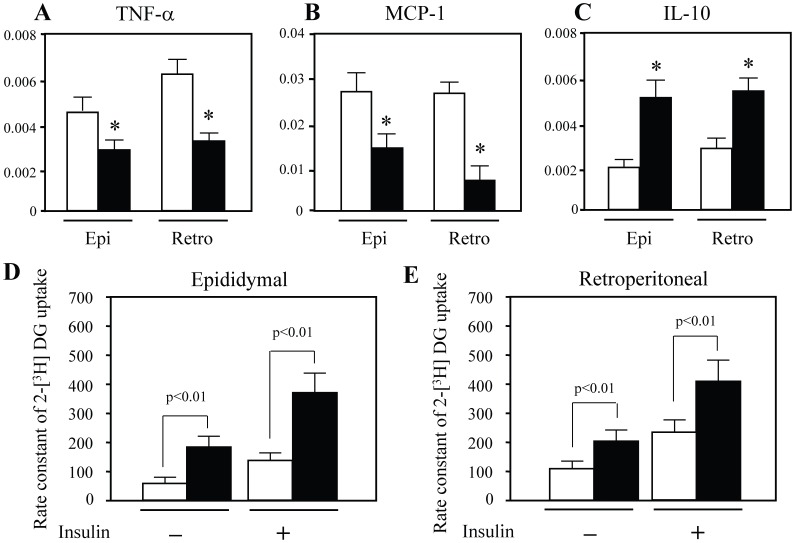
Cytokine levels and glucose uptake in WAT of ATIP1-Tg and WT after treatment with high-cholesterol diet. (**A**) Expression of TNF-α, MCP-1 and IL-10 in epididymal (Epi) and retroperitoneal (Retro) WAT. Open squares; WT, closed squares; ATIP1-Tg. n = 7–8 for each group. *p<0.05 vs. WT. (**B**) Rate constant of 2-[^3^H] DG uptake in epididymal and retroperitoneal WAT was determined with and without insulin (1.0 U/kg) injection. Open squares; WT, closed squares; ATIP1-Tg. n = 6 for each group.

Next, we assessed mRNA levels of proinflammatory cytokines TNF-α and MCP-1, and an immunoregulatory cytokine IL-10 in epididymal and retroperitoneal WAT by RT-PCR. Expression of TNF-α and MCP-1 was markedly decreased in epididymal and retroperitoneal WAT of ATIP1-Tg treated with HCD compared with WT (**Figure 2A**). In contrast, IL-10 expression was significantly increased in ATIP1-Tg (**Figure 2A**). Insulin resistance is closely associated with chronic inflammation, so we examined glucose uptake in WAT using the rate constant of 2-[^3^H]DG uptake. 2-[^3^H]DG uptake was significantly increased in ATIP1-Tg compared with WT, both with and without insulin treatment (**Figure 2B**).

### Reduction of Macrophage Infiltration and Ratio of M1 to M2 Fraction in WAT Macrophages in ATIP1-Tg

**Figure 3:**
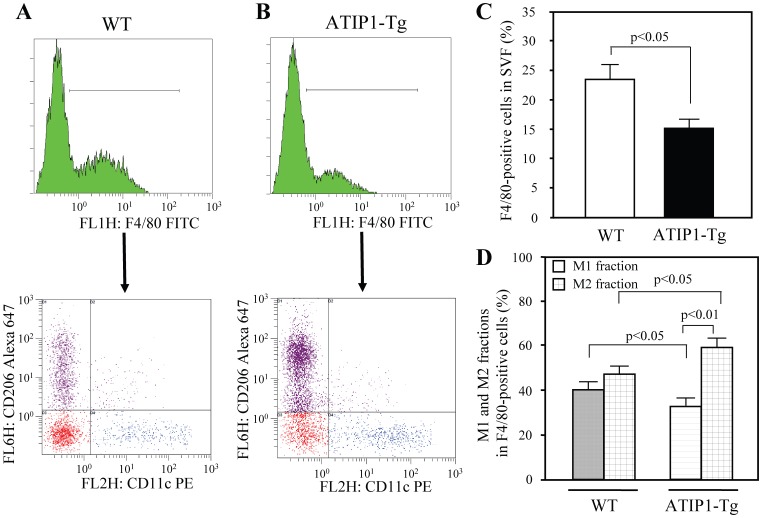
Macrophage infiltration and polarization in ATIP1-Tg and WT after treatment with high-cholesterol diet. Cells in the stromal vascular fraction (SVF) of the epididymal fat pad from each mouse were analyzed by flow cytometry as described in “Methods”. (**A**) Representative results of flow cytometry. F4/80-positive cells were further analyzed with anti-CD11c and anti-CD206 antibodies. Blue dots show M1 macrophages and purple dots show M2 macrophages. Red dots represent both CD11c- and CD206-negative fraction evaluated using isotype controls. (**B**) Percentage of F4/80-positive cells in SVF. n = 5 for each group. (**C**) Ratio of M1 to M2 fraction in F4/80-positive cells. Light gray squares; M1 fraction, dark gray squares; M2 fraction. n = 5 for each group.

In WAT, accumulation of adipose tissue macrophages (ATM) is recognized as an important contributor to the pathogenesis of insulin resistance [11], [21]. Moreover, we observed that mRNA expression of IL-10, an M2-polarized macrophage marker, was increased in WAT in ATIP-Tg, as shown in **Figure 2A**, and therefore we examined the macrophage fractions in WAT. M1 and M2-polarized macrophages were clearly separated by flow cytometry. The macrophage fraction in SVF evaluated by F4/80 staining was significantly smaller in ATIP1-Tg compared with that in WT (16.12%±1.64% in ATIP1-Tg, 24.58±2.96% in WT) (**Figure 3B**). Moreover, ATIP1-Tg exhibited a lower ratio of M1 fraction and a higher ratio of M2 fraction compared to WT (**Figure 3C**).

### Amelioration of Glucose Uptake, Inflammation and Adipocytokine Dysregulation in Type 2 Diabetic Mice Repopulated with BMC Prepared from ATIP1-Tg

ATM are known to be bone marrow-derived [9] and we observed that ATIP1 were highly expressed in BMC isolated from ATIP1-Tg (**Figure S1A**). Therefore, we examined the roles of ATIP1 in hematopoietic cells, focusing on inflammation and glucose uptake of WAT. For this purpose, we generated chimeric mice, ATIP1 chimera (BMC donor; ATIP1-Tg), WT chimera (BMC donor; WT) and KKAy chimera (BMC donor; KKAy), by bulk BMC transplantation using irradiated KKAy as recipients. ATIP1 chimera showed an increase in ATIP1 expression in BMC (**Figure S1B**), indicating successful reconstitution by BMC transplantation.

**Figure 4:**
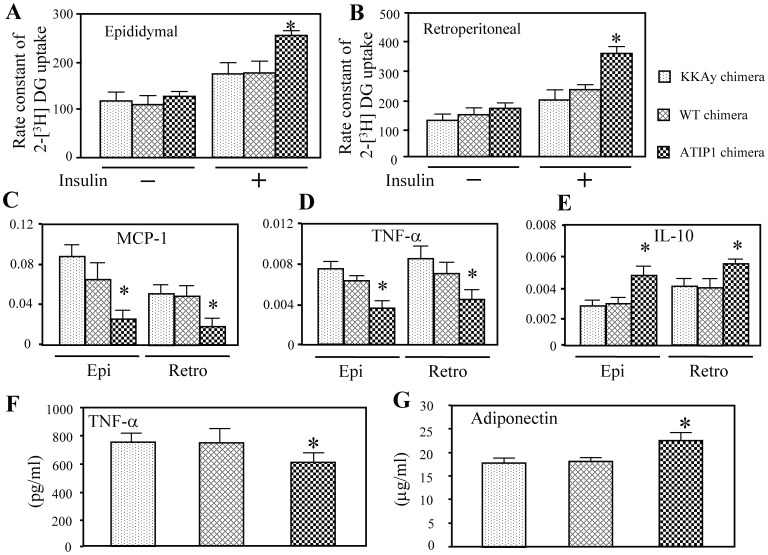
Glucose uptake and cytokines levels in white adipose tissue and serum levels of TNF-α and adiponectin in each chimeric mouse. (A) Rate constant of 2-[^3^H] DG uptake in epididymal (Epi) and retroperitoneal (Retro) WAT were determined with and without insulin (1.0 U/kg) injection. n = 6 for each group. (B) Expression of TNF-α, MCP-1 and IL-10 in epididymal and retroperitoneal WAT. n = 6 for each group. (C) Serum TNF-α and adiponectin concentrations measured by ELISA. n = 8–10 for each group. *p<0.05 vs. KKAy chimera and WT chimera.

The rate constant of 2-[^3^H]DG uptake in the basal condition was similar in both epididymal and retroperitoneal WAT among these three groups of chimera, whereas that in response to insulin was significantly greater in ATIP1 chimera than in other chimeras (**Figure 4A**). Moreover, mRNA expression of MCP-1 and TNF-α was markedly decreased in epididymal and retroperitoneal WAT of ATIP1 chimera compared with other chimeras (**Figure 4B**). In contrast, IL-10 expression was significantly increased in ATIP1 chimera (**Figure 4B**). Although KKAy exhibit a higher level of TNF-α and lower level of adiponectin in serum [22], [23], TNF-α level was decreased and adiponectin level was increased in serum of ATIP1 chimera (**Figure 4C**).

### Reduction of Macrophage Infiltration and Ratio of M1 and M2 Fraction in WAT Macrophages in Type 2 Diabetic Mice Repopulated with BMC Prepared from ATIP1-Tg

**Figure 5:**
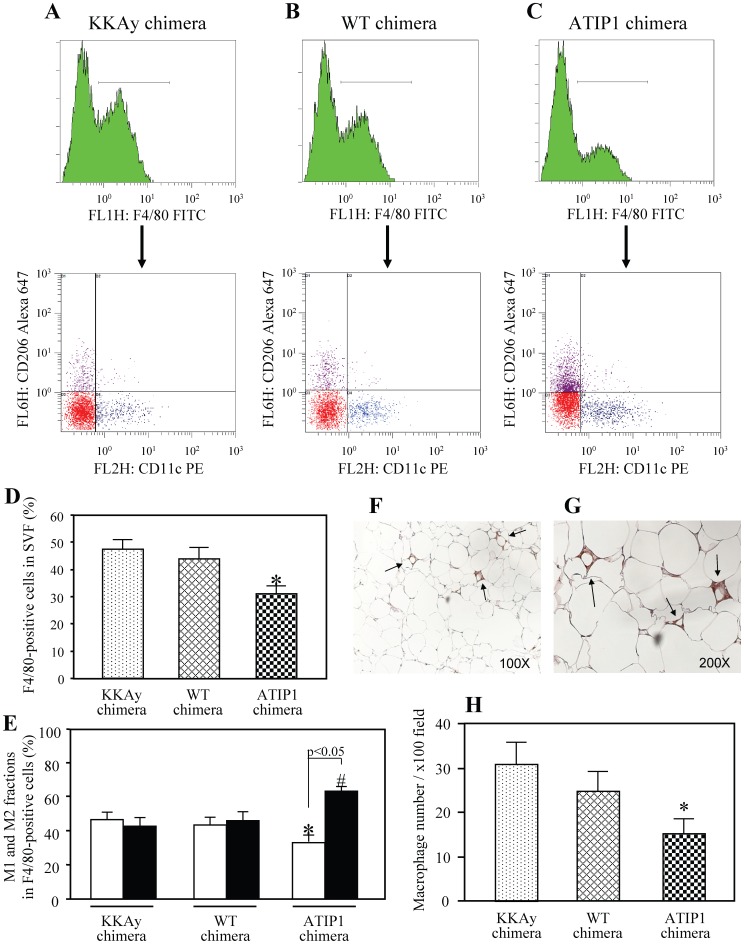
Comparison of macrophage infiltration and polarization in epididymal WAT of chimeric mice. Cells in the stromal vascular fraction (SVF) of the epididymal fat pad from each mouse were analyzed by flow cytometry as described in “Methods”. (**A**) Representative results of flow cytometry. F4/80-positive cells were further analyzed with anti-CD11c and anti-CD206 antibodies. Blue dots show M1 macrophages and purple dots show M2 macrophages. Red dots represent both CD11c- and CD206-negative fraction evaluated using isotype controls. (**B**) Percentage of F4/80-positive cells in SVF. n = 5 for each group. *p<0.05 vs. KKAy chimera and WT chimera. (**C**) Ratio of M1 and M2 fraction in F4/80-positive cells. Open squares; M1 fraction, closed squares; M2 fraction. *p<0.05 vs. M1 fraction in KKAy chimera and WT chimera, ^#^p<0.05 vs. M2 fraction in KKAy chimera and WT chimera. n = 5 for each group. (**D**) Immunohistochemical staining of F4/80 in epididymal WAT at low (×100) and high (×200) magnification. Sections from epididymal fat pads of chimeric mice were stained with anti-F4/80 antibodies, followed by DAB staining. F4/80-positive cells are observed as localized macrophage clusters (arrow). Histogram of macrophage number per field at ×100 magnification is shown in the lower panel. n = 6 for each group. *p<0.05 vs. KKAy chimera and WT chimera.

To examine whether the reduced inflammation in WAT of ATIP1 chimera was correlated with ATM, we also performed flow cytometry to detect M1 and M2 macrophages in F4-80-positive cells from the isolated SVF (**Figure 5A**). ATIP1 chimera displayed a smaller F4/80-positive cell fraction in SVF (32.40±2.46%) than that in WT chimera (44.18% ±4.08%) and KKAy chimera (47.92% ±3.15%) (**Figure 5B**). Moreover, in F4/80-positive cells isolated from SVF, M1-polarized macrophages were decreased and M2-polarized macrophages were increased in ATIP1 chimera compared with other chimeric mice (**Figure 5C**). Immunohistochemical staining of F4/80 in epididymal fat pads showed localized macrophage clusters surrounding adipocytes (**Figure 5D**). Consistent with the results of flow cytometry, the number of F4/80-positive macrophages per field at ×100 magnification was significantly decreased in ATIP1 chimera (**Figure 5D**).

## Discussion

The present study demonstrated that ATIP1 could play an important role in HCD-induced inflammation and insulin resistance in WAT through the attenuation of macrophage infiltration and M1 polarization, leading us to further explore the roles of ATIP1 in hematopoietic cells in the pathogenesis of insulin resistance, focusing on WAT. Accordingly, we observed that transplantation of ATIP1-overexpressing BMC improved inflammation and consequently ameliorated insulin resistance in WAT of type 2 diabetic and obese KKAy as the recipient. Macrophage accumulation in epididymal WAT and polarization toward an M1 state were reduced in ATIP1 chimeric mice. These findings suggest that ATIP1 in hematopoietic cells has a crucial role in amelioration of inflammation in WAT via macrophage infiltration and polarization.

Obesity is an early step in the progression of metabolic syndrome and type 2 diabetes [24]. Moreover, increasing evidence has suggested that low-grade inflammation with infiltration of inflammatory cells in WAT is closely associated with obesity [25], resulting in insulin resistance in WAT, which plays an important role in the onset and progression of type 2 diabetes [25], [26]. Although the precise cell types and detailed mechanisms involved in this process are not fully understood, ATM are known to be an important contributor to inflammation followed by insulin resistance [27], [28]. M1 ATM express proinflammatory cytokines such as TNF-α and IL-6, while M2 ATM are reported to express the chitinase family member Ym1, the anti-inflammatory cytokine IL-10, arginase and surface lectins Mgl1 and Mgl2 [29], [30]. According to previous reports, ATM isolated from WAT of lean animals expressed hallmarks of polarization toward an M2 state, suggesting a potentially beneficial function of these cells, whereas in diet-induced obese animals, the number of M1 macrophages and the ratio of M1 to M2 macrophages were increased in WAT [13], [14], [31]. TNF-α, secreted from enlarged adipocytes, stimulates preadipocytes and endothelial cells to produce MCP-1 [32], then the increased MCP-1 recruits monocytes into the adipose tissue and stimulates M1 ATM differentiation [13], [28]. Once macrophage activation and infiltration are initiated, they secrete numerous cytokines and chemokines to further activate themselves [12]. These feedback loops increasingly impair insulin signaling in adipocytes, eventually leading to massive adipocyte lipolysis, necrosis and systemic insulin resistance [11]. Based on our results, we speculate that ATIP1 may inhibit such feedback loops via attenuation of HCD- or obesity-induced inflammation.

Based on analysis of annotated functions of genes correlated with body mass, transcripts characteristic of macrophages were observed to be coordinately upregulated in direct proportion to body weight in several models of obesity [33], [34]. This observation shows that macrophage infiltration in adipose tissue might be positively correlated with adiposity. In ATIP1-Tg mice with HCD, WAT weight and adipocyte size were decreased compared with those in WT controls (**Figure 1A and 1B**). Moreover, HCD-induced ATM infiltration and M1 differentiation were ameliorated in ATIP1-Tg mice (**Figure 3B**), with suppressed inflammation in WAT, supporting the possibility that ATM are activated in an adipocyte-dependent manner.

Weisberg et al. demonstrated that ATM were derived from bone marrow precursors that migrate from the peripheral circulation [9]. We observed that ATIP1 were highly expressed in BMC isolated from ATIP1-Tg mice (**Figure S1A**). The BMC transplantation approach has been expected to be a crucial technique in approaching the pathogenesis of metabolic disorders [35]. Saberi et al. showed that hematopoietic cell-specific deletion of Toll-like receptor 4 ameliorates adipose tissue insulin resistance in HCD-fed mice, using BMC-transplanted chimeric mice [36]. Cudejko et al. demonstrated that p16INK4a deficiency modulates macrophage polarization, using BMC chimeric mice [37]. Accordingly, we performed bone marrow transplantation of ATIP1-Tg using KKAy mice as recipients. Interestingly, ATIP1 chimera showed reduced ATM infiltration and M1 polarization, associated with amelioration of inflammation and improvement of insulin sensitivity in WAT (**Figures 4** **and** **5**). These observations support the idea that ATIP1-overexpressing ATM are less activated in obese mice and type 2 diabetic mice, which, in turn, could attenuate inflammation and enhance insulin sensitivity in WAT. Therefore, we speculate that the amelioration of ATM activation in ATIP1-Tg mice is at least in part due to the overexpression of ATIP1 in ATM.

Macrophages are mononuclear phagocytes and ATM migrate from circulating monocytes. MCP-1 and its receptor C-C motif chemokine receptor 2 (CCR2) are known to play an important role in monocyte migration to peripheral tissues [38], [39]. Mouse monocytes can be divided into two subsets: CX3CR1^high^CCR2^−^Ly-6C^low^ (CCR2^−^) monocytes and CX3CR1^low^CCR2^+^Ly-6C^high^ (CCR2^+^) monocytes [40], [41]. CCR2^−^ monocytes accumulate in noninflamed tissues and are termed “resident”, while CCR2^+^ monocytes are potent inflammatory mediators with higher capacity for migration into inflamed tissues [42], [43]. Consistent with this theory, Lumeng et al. demonstrated that in lean WAT, resident ATM are polarized toward an M2 state derived from CCR2^−^ monocytes, whereas obesity leads to an increase in circulating CCR2^+^ monocytes, which are the source of the infiltrating M1 macrophages in obese WAT [13]. In our study, HCD-induced M1 polarization was reduced in ATIP1-Tg mice (**Figure 3**); however, there was no significant difference in ATM content between ATIP1-Tg and WT when they were fed a normal diet (**Figure S2A, S2B and S2C**). Therefore, we assumed that ATIP1 would not have significant effects on resident CCR2^−^ monocytes/M2 ATM, but would suppress the increase in CCR2^+^ monocytes/M1 ATM related to the MCP-1/CCR2 axis. Further studies will be needed to elucidate the mechanisms by which ATIP1 regulates the MCP-1/CCR2 signaling cascade.

Transcriptional regulation of macrophage polarization has been extensively studied. For example, M1 macrophage polarization involves the activation of a set of transcription factors, such as NF-κB, AP-1, C/EBPb, PU.1 and IFN-regulatory factors (IRFs) [44], [45]. On the other hand, M2 macrophage polarization involves the activation of a set of transcription factors, such as STAT6 and peroxisome proliferator-activated receptor (PPAR) γ [29], [46]. Moreover, recent studies have revealed that epigenetic regulation also plays an important role in macrophage polarization [47]. In the present study, we have not investigated the detailed mechanism of ATIP1-induced M2 macrophage polarization; however, our recent study demonstrated that AT_2_ receptor stimulation ameliorated insulin resistance in diabetic mice, associated with PPARγ activation [48], and there are relations between IRFs and the AT_2_ receptor [49], [50]. Therefore, we are investigating whether ATIP1-induced transcriptional regulation such as PPARγ activation would be involved in this mechanism. ATIP1 is known to localize in either the mitochondria [3] or Golgi [2], as well as the cell membrane; therefore, ATIP1 is considered to be a microtubule-associated protein [51]. Although the functions of ATIP associated with microtubules are totally unknown, microtubule-targeting agents including PPARγ inhibitors such as GW9662 are a very successful class of anti-cancer drugs with therapeutic benefits in both hematopoietic and solid tumors [52], [53]. Potential roles of microtubule-associated protein in macrophage polarization still remain an enigma; however, non-binding of ATIP with the AT_2_ receptor around microtubules could act in transcriptional regulation involving PPARγ etc.

Sica et al. reviewed the recent research on macrophage polarization in pathological conditions [54]. M2 macrophages play mainly a beneficial role in obesity, metabolism, inflammation and tissue repair; however, allergy is associated with M2 polarization of macrophages [55]. Moreover, tumor progression is associated with a phenotype switch from M1 to M2 at least in some models of carcinogenesis in the mouse [56]. ATIP1 is a mitochondrial protein that acts as a tumor suppressor [57]. ATIP1-induced M2 macrophage polarization may be a contradictory function in tumor pathogenesis. We speculate that an abnormal switch from M1 to M2 would inhibit M1 macrophage-induced antitumor activity of T cell-mediated elimination and equilibrium phases during tumor progression induced by cancer-related inflammation; however, originally ATIP1 could prevent progression of cancer itself. Further investigation is needed to assess the functional effect of ATIP1 on these balances between cancer cell progression and anti-tumor effects by the recruited cells surrounding the tumor.

We previously reported that over-expression of ATIP1 improves vascular remodeling independent of the AT_2_ receptor in inflammation-induced vascular injury [6]. We cannot exclude the involvement of AT_2_ receptor activation in the decrease of inflammation and improvement of insulin resistance in WAT. We tried to generate “AT_2_ receptor-deleted chimeric mice” (BM donor: AT_2_ receptor-deficient mice); however, this chimeric mouse was lethal and we could not analyze the effect of the AT_2_ receptor in this set of experiments. Moreover, using an AT_2_ receptor antagonist such as PD123319 is not appropriate in this study, because PD123319 has a short half-life, and its permeability and the appropriate concentration for adipose tissue are totally unknown. We speculate that deletion of the AT_2_ receptor reduced BM engraftment due to less differentiation activity, since the AT_2_ receptor is known to play an important role in cellular differentiation [58], [59], [60]. Therefore, we are not able to conclude whether the effect of ATIP on macrophage differentiation is dependent or independent of AT_2_ receptor signaling. To address the involvement of the AT_2_ receptor in ATIP-induced modulation of macrophage polarization, more detailed analysis should be performed in the future. It is also possible that the metabolic difference of adipose tissue between ATIP1-Tg and WT might result from differences of ATIP1 expression in adipose tissue and other organs, also independent of inflammation. This issue remains to be addressed.

In summary, we expect ATIP1 could be a novel therapeutic target to prevent and treat metabolic disorders via innate immunity, with a reduction in macrophage infiltration and a change in the ratio of M1 to M2 macrophages in WAT. Moreover, further elucidation of the functional regulation of ATIP1, including investigating phosphorylation and dephosphorylation and transcriptional control, and finding possible ligands should be performed.

## Supporting Information

Figure S1 **ATIP1 protein level in BMC.** BMC were isolated from bilateral tibias, femurs and iliac bones, and protein level was measured by immunoblotting with an antibody against ATIP1. (**A**) ATIP1 protein level in BMC of ATIP1-Tg and WT. (**B**) ATIP1 protein level in BMC of KKAy chimera, WT chimera and ATIP1 chimera. Total protein was prepared from BMC isolated from six crushed bones (bilateral tibias, femurs, and iliac bones). Immunoblot analysis was performed using rabbit anti-mouse ATIP1 polyclonal antibody (kindly provided by Dr. C. Nahmias, Inserm, U1016, France) and a secondary anti-rabbit antibody (Cell Signaling Technology, Inc, Danvers, MA).
(TIF)

Figure S2 **Comparison of macrophage polarization in epididymal WAT of ATIP1-Tg and WT fed normal diet (ND).** Cells in the stromal vascular fraction (SVF) of the epididymal fat pad from mice fed ND for 18 weeks were analyzed by flow cytometry as described in “Methods”. (**A**) Representative results of flow cytometry. F4/80-positive cells were further analyzed with anti-CD11c and anti-CD206 antibodies. Blue dots show M1 macrophages and purple dots show M2 macrophages. Red dots represent both CD11c- and CD206-negative fraction evaluated using isotype controls. (**B**) Percentage of F4/80-positive cells in SVF. n = 5 for each group. (**C**) Ratio of M1 to M2 fraction in F4/80-positive cells. Light gray squares; M1 fraction, dark gray squares; M2 fraction. n = 5 for each group.
(TIF)
