# Supplementary figures and images for: Effect of Angiotensin II Type 2 Receptor-Interacting Protein on Adipose Tissue Function via Modulation of Macrophage Polarization
Source: PLoS One. 2013 Apr 2;8(4):e60067. doi: 10.1371/journal.pone.0060067 (PMC3614946; doi:10.1371/journal.pone.0060067)

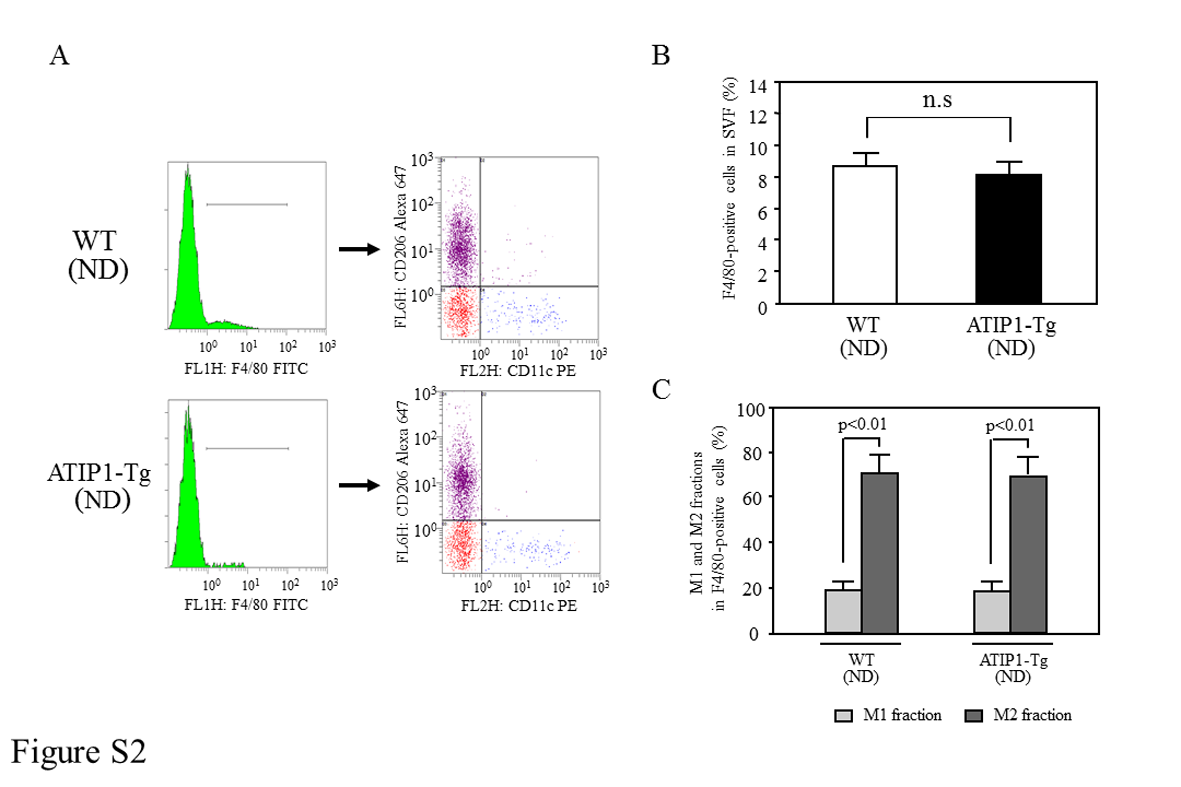

Supplement: Figure S2 — Comparison of macrophage polarization in epididymal WAT of ATIP1-Tg and WT fed normal diet (ND). Cells in the stromal vascular fraction (SVF) of the epididymal fat pad from mice fed ND for 18 weeks were analyzed by flow cytometry as described in “Methods”. (A) Representative results of flow cytometry. F4/80-positive cells were further analyzed with anti-CD11c and anti-CD206 antibodies. Blue dots show M1 macrophages and purple dots show M2 macrophages. Red dots represent both CD11c- and CD206-negative fraction evaluated using isotype controls. (B) Percentage of F4/80-positive cells in SVF. n = 5 for each group. (C) Ratio of M1 to M2 fraction in F4/80-positive cells. Light gray squares; M1 fraction, dark gray squares; M2 fraction. n = 5 for each group. (TIF) [file pone.0060067.s002.tif]
